# Complete Genome Sequences of Four Streptococcus parasuis Strains Obtained from Saliva of Domestic Pigs in Japan

**DOI:** 10.1128/mra.01245-21

**Published:** 2022-02-17

**Authors:** Ryohei Nomoto, Kasumi Ishida-Kuroki, Ichiro Nakagawa, Tsutomu Sekizaki

**Affiliations:** a Department of Infectious Diseases, Kobe Institute of Health, Kobe, Japan; b Antimicrobial Resistance Research Center, National Institute of Infectious Diseases, Tokyo, Japan; c Department of Microbiology, Graduate School of Medicine, Kyoto University, Kyoto, Japan; d Graduate School of Agricultural and Life Sciences, The University of Tokyo, Tokyo, Japan; University of Maryland School of Medicine

## Abstract

Streptococcus parasuis is a close relative of Streptococcus suis, an important zoonotic pathogen that causes various diseases in pigs and humans. Here, we report the complete genome sequences of four strains, including the type strain of *S. parasuis*, isolated from the saliva of healthy pigs in Japan.

## ANNOUNCEMENT

Streptococcus parasuis was formerly classified as serotype 20, 22, and 26 of the zoonotic pathogen Streptococcus suis and has recently been taxonomically separated from S. suis as a novel species (1). Since it has been proposed as a novel species, the biological and pathological features of *S. parasuis* have remained unclear because of the lack of whole genetic information on *S. parasuis*. Here, we report the complete genome sequence of four *S. parasuis* strains, SUT-7, SUT-286^T^, SUT-380, and SUT-503, which were previously isolated by our group from the saliva of clinically healthy pigs in Ibaraki Prefecture, Japan (1, 2).

The four *S. parasuis* strains were cultured on Todd-Hewitt agar at 37°C for 24 h under 5% CO_2_. Genomic DNA was extracted using the PureLink genomic DNA minikit (Thermo Fisher Scientific, USA), according to the manufacturer’s protocol. For Illumina sequencing, genomic libraries were prepared using the Nextera XT DNA library prep kit (Illumina, USA), and sequencing was performed using the Illumina MiSeq system with v2 chemistry (2 × 250-bp format). The raw reads were quality filtered and trimmed using fastp v0.23.2 (3) with default settings. Library preparation for Oxford Nanopore Technologies (ONT) sequencing followed the rapid barcoding DNA sequencing protocol with the SQK-RBK004 kit (ONT, UK) without DNA size selection, and the libraries were sequenced using a single R9.4.1/FLO-MIN106 flow cell on a MinION sequencer (ONT). Base calling was performed using Guppy v3.0.7 in “accurate” mode implemented on the MinIT device (ONT). The ONT raw reads were demultiplexed, and ONT adapters were trimmed using Porechop v0.2.4 (https://github.com/rrwick/Porechop). The numbers of reads are listed in Table 1.

**TABLE 1:** Assembly metrics and annotated features of four Streptococcus parasuis strains isolated from the saliva of pigs

Strain	Yr of isolation	Genome size (bp)	Plasmid size (bp)	No. of contigs	No. of Illumina reads	No. of Nanopore reads	G+C content (%)	Total no. of CDSs^*a*^	*N*_50_ of Nanopore reads (bp)	GenBank accession no.	DRA accession no.
SUT-7	2012	2,202,836		1	1,024,534	37,584	39.9	2,132	8,640	AP024275	DRR332823, DRR332831
SUT-286^T^	2013	2,197,342		1	914,654	28,154	40.0	2,138	9,226	AP024276	DRR332824, DRR332832
SUT-380	2013	2,109,881	25,027; 6,937	3 (including plasmid)	827,158	27,017	39.8	2,130	7,605	AP024277, AP024278,^*b*^ AP024279^*b*^	DRR332826, DRR332834
SUT-503	2014	2,065,066		1	882,810	20,835	39.9	2,053	7,116	AP024280	DRR332827, DRR332835

a CDSs, coding DNA sequences.

b A complete plasmid sequence.

Hybrid assemblies with the ONT and Illumina data were performed using the Unicycler v0.4.8 pipeline (4) with default settings. The Illumina reads were assembled using SPAdes v3.13.0 (5), and the resulting long-anchor contigs were assembled with the ONT reads using an optimized version of miniasm (6) and Racon v1.4.3 (7). Pilon v1.23 (8) was used within Unicycler to iteratively polish the assembly with Illumina reads. The circularity of each contig was confirmed using the Unicycler log files. The circularized genome was rotated to the default starting gene, *dnaA*. The chromosome and plasmid sequences were annotated using the DDBJ Fast Annotation and Submission Tool (DFAST) (9). The assembly metrics and annotation features are shown in Table 1.

### Data availability.

The complete genome sequences and raw sequence data of the four strains were deposited in DDBJ/EMBL/GenBank under BioProject accession no. PRJDB10858. The accession numbers of the complete genome sequences are AP024275, AP024276, AP024277, AP024278 (plasmid sequence of SUT-380), AP024279 (plasmid sequence of SUT-380), and AP024280. The DRA accession numbers are DRR332823, DRR332824, DRR332826, and DRR332827 (Illumina read data) and DRR332831, DRR332832, DRR332834, and DRR332835 (ONT read data).
